# Functional tissue units and their primary tissue motifs in multi-scale physiology

**DOI:** 10.1186/2041-1480-4-22

**Published:** 2013-10-08

**Authors:** Bernard de Bono, Pierre Grenon, Richard Baldock, Peter Hunter

**Affiliations:** 1 Auckland Bioengineering Institute, University of Auckland, Symonds Street, Auckland 1010, New Zealand; 2 CHIME Institute, Archway Campus, University College London, London, UK; 3 European Bioinformatics Institute, Cambridge, UK; 4 MRC Human Genetics Unit, MRC IGMM, University of Edinburgh, Edinburgh, UK

## Abstract

**Background:**

Histology information management relies on complex knowledge derived from morphological tissue analyses. These approaches have not significantly facilitated the general integration of tissue- and molecular-level knowledge across the board in support of a systematic classification of tissue function, as well as the coherent multi-scale study of physiology. Our work aims to support directly these integrative goals.

**Results:**

We describe, for the first time, the precise biophysical and topological characteristics of functional units of tissue. Such a unit consists of a three-dimensional block of cells centred around a capillary, such that each cell in this block is within diffusion distance from any other cell in the same block. We refer to this block as a functional tissue unit. As a means of simplifying the knowledge representation of this unit, and rendering this knowledge more amenable to automated reasoning and classification, we developed a simple descriptor of its cellular content and anatomical location, which we refer to as a primary tissue motif. In particular, a primary motif captures the set of cellular participants of diffusion-mediated interactions brokered by secreted products to create a tissue-level molecular network.

**Conclusions:**

Multi-organ communication, therefore, may be interpreted in terms of interactions between molecular networks housed by interconnected functional tissue units. By extension, a functional picture of an organ, or its tissue components, may be rationally assembled using a collection of these functional tissue units as building blocks. In our work, we outline the biophysical rationale for a rigorous definition of a unit of functional tissue organization, and demonstrate the application of primary motifs in tissue classification. In so doing, we acknowledge (i) the fundamental role of capillaries in directing and radically informing tissue architecture, as well as (ii) the importance of taking into full account the critical influence of neighbouring cellular environments when studying complex developmental and pathological phenomena.

## Background

Current standards and practices in histology information management rely heavily on implicitly represented knowledge derived from morphological analyses of two-dimensional images captured from tissue samples. These practices have not significantly facilitated the integration of tissue- and molecular-level knowledge across the board in support of a systematic classification of tissue function, as well as the multi-scale study of physiology.

This work discusses one particular element in an overall system of ontology for physiology in multicellular organisms in support of these integrative goals. Specifically, this paper describes for the first time the precise biophysical and topological characteristics of a generic functional unit in tissues. Such a unit consists of a three-dimensional block of cells centred around a capillary, such that each cell in this block is within diffusion distance from any other cell in the same block. We refer to this block as a "**functional tissue unit**" (**FTU**). As a means of simplifying the knowledge representation of an FTU, and rendering this knowledge more amenable to automated reasoning, we developed a simple descriptor of the cellular content and anatomical location of an FTU, which we refer to as a "**primary tissue motif**" (**PTM**). In this work we demonstrate the application of PTM knowledge in tissue classification.

Given that the spatial characteristics of an FTU are constrained to ensure the homogeneous diffusive admixture of extracellular molecules across its entire volume, including those molecules that enter or leave the FTU by advective means (*e.g.* via the blood supply), the rest of this section will briefly introduce the key biophysical constraints on molecular transport processes as a means to explain the central, rate limiting, role that tissues fulfill in transport physiology.

### Overview of transport physiology

In protein biology, tissues serve two key functions, namely, to (i) support the synthesis and folding of proteins within cells, and (ii) to regulate the extent by which proteins are allowed to meet other molecules across compartments, thus facilitating or inhibiting the fulfillment of protein function. A metabolically critical goal for transport physiology is the regulation of the extent by which proteins are allowed to encounter H^+^ ions [1], in view of the crucial role these ions play in the protein folding process. A second key goal for transport processes is to convey molecule-mediated communication between cells, through juxtacrine (*e.g.* Notch signaling [2]), paracrine (*e.g.* IGF-1 [3]), endocrine (*e.g.* thyroid hormone action [4]) and exocrine (*e.g.* immunological factors passed on via lactation [5]) modalities. This type of communication serves to co-ordinate cells on protein production and the intercompartmental trafficking of molecules.

Given the above physiological goals, the generic architecture of cellular arrangement within any type of solid tissue (*e.g.* skeletal muscle, liver, brain, lung, *etc.*) has to ensure that the core processes of (i) metabolism and (ii) communication achieving these goals are fulfilled.

In this paper, a first step in interpreting tissue structure in this key is taken by identifying and characterizing a basic unit of tissue that fulfills the above process goals (*i.e.* metabolism and communication). This continuous block of tissue, known as a functional tissue unit, ensures the viability of its cellular content by satisfying the biophysical constraints for these goals to be reached.

## Results

### Physiological and biophysical characterisation of an FTU

Given the metabolic goals discussed above, a core biophysical requirement is that cells have to be within diffusion distance of at least one capillary blood vessel to secure appropriate rates of (i) delivery of supplies (*e.g.* oxygen, glucose) and (ii) elimination of waste (*e.g.* carbon dioxide, H^+^, urea). As the maximum distance over which diffusion occurs is approximately 100 μm, most mammalian cells in solid tissues are found within 50 μm of a capillary [6].

The same 100-μm diffusion limit constraint applies to any local molecular exchange between cells (*e.g.* electrolytes, metabolic intermediates, messenger molecules, *etc.*). For instance, the paracrine communication modality, which enables the creation of gene expression regulatory networks that span across neighbouring cell types in the same tissue (*e.g.* see refs [7,8]), is also bound by the same distance constraint.

**Figure 1:**
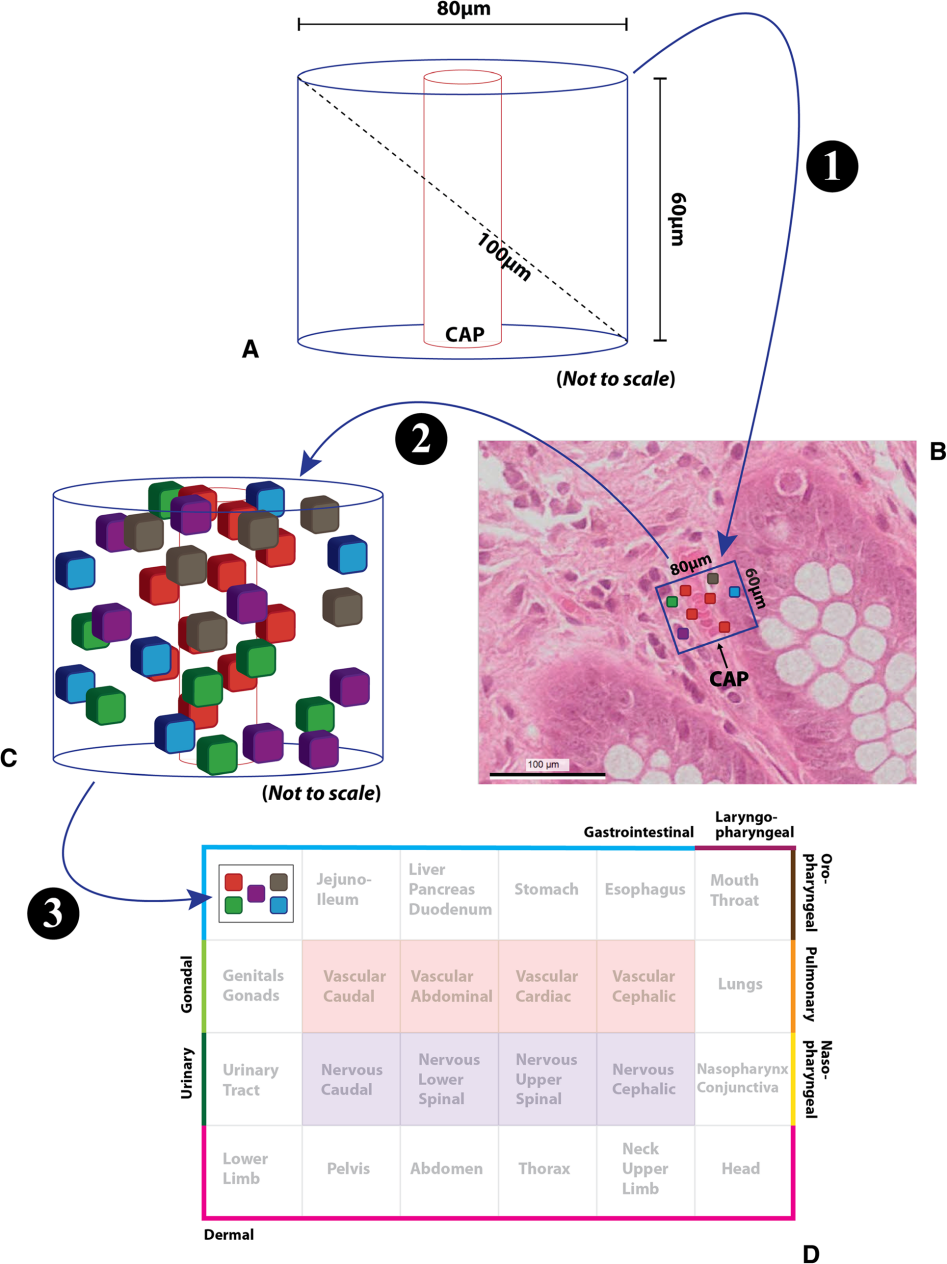
**Example workflow illustrating the acquisition and processing of FTU data from a three-dimensional reconstruction of human colon tissue.** Step 1: The FTU template **(A)** is prepared according to the biophysical constraints under consideration, such that the long axis of the resulting cylindrical block of tissue is that of the feeding capillary (CAP) on which it is metabolically dependent. This template is applied to an appropriate volumetric region in the three-dimensional histology image dataset **(B)**. The various cells within this region (coloured boxes) are typed and their position recorded (*Note*: red boxes represent endothelial cells, here shown lining the feeding capillary – CAP – and the erythrocytes within its lumen). Step 2: The cellular annotations across the full extent of the FTU cylindrical boundaries **(C)** are stored, together with the image data and the anatomical provenance of the tissue sample. Step 3: As the resulting primary tissue motif for the above colonic FTU uses standard reference ontology terms to represent both (i) a non-redundant list of distinct cell types, as well as (ii) the anatomical region of origin for the sourced tissue material, a terse graphical depiction of the constitution of this FTU may be automatically included in the context of whole-body anatomy maps, such as the one schematized by the ApiNATOMY tool [9] in **(D)**. In this schematic, the outer boundary of the map represents the various epithelial surface categories (each individually coloured and labelled), and the inside tiles represent vascular (red) and neural (purple) structures respectively.

It is therefore possible to identify a unit of solid tissue consisting of a well-defined heterogeneous set of cells that is both (i) metabolically dependent on the same capillary, as well as (ii) the cellular substrate for tissue-level molecular pathways co-ordinated via paracrine communication. This block of tissue has a cylindrical shape whose long axis is that of the feeding capillary on which it is metabolically dependent (Figure 1A). The rigid biophysical constraint that within this cylinder no two cells may be more than 100 μm apart (dashed line, Figure 1A) provides a fundamental mathematical limit to calculate the absolute dimensions of an FTU. As the application of this limit in isolation gives rise to multiple solutions, the addition of a second constraint is required to provide specific values to the diameter and length of a cylindrical tissue block. For instance, by requiring that the particular cylinder of choice is one that achieves the maximal volume (*i.e.* the volume ensuring the greatest possible mass of protein metabolic machinery within the FTU substrate) we have estimated the diameter of that cylindrical block to be about 80 μm and the length approximately 60 μm.

### Representing FTU-derived knowledge as a primary tissue motif

As three-dimensional (3D) image data about FTUs is anticipated to be complex, it is critical that computationally efficient approaches are developed to represent salient biological knowledge about FTUs. To this end, we developed the notion of a primary tissue motif, derived from FTU information, in support of tissue classification and cross-scale integrative goals.

If descriptions of FTUs are to tersely provide information about cells that are clustered together (Figure 1C) to locally support a tissue-level molecular pathway, then the minimal FTU-derived knowledge required should include:

i. a non-redundant list of distinct cell types (graphically represented in Figure 1D as a set of five differently-coloured cells in a box), observed in an FTU’s 3D image data, to indicate the kind of cell that can contribute extracellular molecules to the local tissue-level network through diffusion, as well as

ii. the anatomical region of origin for the sourced tissue material of the FTU (represented in Figure 1D through the nesting of the above set of 5 cells within the colonic region of the body map) as a means to identify the advective routes through which body-level molecular networks can interlink with the local pathways.

The formal representation of the above minimal knowledge, in the form of a PTM, provides a surrogate avenue to automating and quantifying the comparison of FTUs as a means to classifying tissues in terms of well-defined functional criteria.

Part 1 of the Methods Section briefly elicits a number of requirements for such knowledge representation and overviews the application of the Basic Formal Ontology (BFO) [10] to the approach taken. In this section we present a preliminary specification of the core concepts and, in the section that follows, draw on these elements to illustrate how this framework could lead to the classification of FTUs.

Tissues are made of cells and extracellular matrix. In the present paper, we leave extracellular matrix constituents of tissues aside and focus on cellular constitution. Anatomically, different types of tissues demonstrate regularity in cellular composition and organisation. For a given tissue, specific patterns of cell types and the organisation of cells of these different types are characteristics. These patterns are what we call tissue motifs. Without providing a definition of a specific tissue motif, we can register that the constraints present two elements: (i) the types of cell in a tissue, (ii) the organisation of cells (of different or the same types) in a tissue. When focusing, in the present paper, on primary tissue motifs we attend to elements of the first type, that is to say, the list of types of cells constituting a functional tissue unit.

Definition. FTU **u** *is defined by***u** is a functional tissue unit, *i.e.* a three-dimensional, maximally connected, block of cells centred around a capillary, such that each cell in this block is within diffusion distance from any other cell in the same block. FTU is a subtype of BFO’s material object (we leave open whether it falls more specifically under one of the subtypes of material objects, namely: object, aggregate of object or fiat part of object).

Definition. containsCell **u v** *is defined by***u** is a FTU which has cell **v** as a part (containsCell is a specialisation of the "has part" relation).

Definition. containsCellType **u v** *is defined by***u** is a functional tissue unit such that there is at least one cell of type **v** in the containsCell relation to **u** (containsCellType is a relation between an instance and a type).

A primary tissue motif can be defined as an extensional set theoretical abstraction over the containsCellType, *i.e.* as the set of cell types entering in the containsCellType relation with a given FTU. For a given FTU, then, its primary tissue motif is unique. For convenience, we use the functional notation PTM(**u**) to denote the primary motif of a functional tissue unit **u**. Using set theoretical notation, we can then write:

$$\text{PTM}\left(u\right)\;\text{is}\;\text{defined}\;\text{by}\;\left\{x/\text{containsCellType}\;u\;x\right\}$$

Primary tissue motifs are but one aspect of tissue motifs. Consistently with BFO, we do not include set theoretical constructs in the ontology. However, this does not prevent us from using the formal apparatus of set theory in order to record PTMs. We leave open whether PTMs are entities in their own rights or abstractions overs Tissue Motifs.

Finally, we give ourselves the means to formally register the anatomical location of an FTU so as to record the fact that the FTU belongs to, say, liver tissue or any other anatomical location. To this end, we use the relation anatomicalLocation linking an FTU to an anatomical location such as represented in the Foundational Model of Anatomy [11].

The foregoing is enough for the current purpose and to illustrate the annotation of FTU data in the form of histological images (as exemplified in part 2 of the Methods Section). Images are one kind of representation for FTUs - images constitute imaging data. To these images, we can attach records of formal descriptions of the FTUs depicted. Here, such formal descriptions amount to the annotation of image elements (which depict FTU parts) with the kind of entity they instantiate. In our case, the image elements are of cells and the kind of entity to which they are associated are cell types (for which we use the CellType ontology). Annotation links are registered using the containsCellType relation introduced above.

On the basis of the foregoing, and with the provision of data regarding the cellular composition of FTU instances, we can also illustrate the prospective approach to the classification of FTUs, here on the basis of their primary tissue motifs. The classification of FTUs consists in fleshing out a taxonomy of FTUs. There are, of course, many ways of classifying FTUs. The present discussion is concerned with the classification of FTUs on the basis of their PTMs.

For two given FTUs, **x** and **y**, we can define the following relation of PTM-equivalence which holds when the PTMs of **x** and **y** are the same:

$$\text{ptm}-\text{equivalence}\;\left(x,y\right)\;\text{is}\;\text{defined}\;\text{by}\;\text{PTM}\left(x\right)=\text{PTM}\left(y\right).$$

If PTMs are handled set-theoretically, the relation holds whenever the PTMs have the same members: ptm-equivalence (**x**,**y**) *if and only if* for all **z**, (containsCellType **x z**) is equivalent to (containsCellType **y z**).

In some cases, only some of the cell types contained in one FTU are contained in the other. When the first contains no other cell type, we say that **x** is ptm-subsumed by **y** (alternatively, **y** ptm-subsumes **x**). When, however, **x** contains other cell types than those contained by **y**, we say that **x** and **y** ptm-overlap.

Using these relations, bearing in mind they can be read as standard set-theoretic relations, it becomes possible to consider unions of PTMs and, thus, complex FTUs that may be broken down into unifying multiple PTMs. This route leads to the definability of aspects of tissues, including organs, as aggregates of cells. The outcome is a theory of parts and wholes among tissues that may be used in the characterisation of complex tissues using FTUs and PTMs and the recording of varied tissue types. Using these tools, it becomes possible to flesh out PTMs, FTUs, and tissue classification systems. The required theory remains an item for future work.

In the next section, we exemplify how to proceed with classification based on semantic similarity between the elements of PTMs corresponding to recorded FTUs. In addition, part 2 of the Methods section overviews the process of 3D image annotation as a means to show how the above representation can be used to register knowledge about FTUs.

### Classifying primary tissue motifs

Primary tissue motifs take into account the cellular constituents in an FTU. Consequently, one avenue to objectively compare FTUs is through the calculation of pairwise similarity of their primary motif surrogates in an approach that is, in principle, similar to primary sequence comparison in protein structure classification. In terms of this analogy between pairwise comparisons of amino acid and cellular constituents of biological structures, semantic similarity matrices (*e.g.*[12]) for CellType ontology terms in PTMs take on the role of amino acid substitution matrices in primary protein sequence alignment (*e.g.*[13-15]).

In this section, we describe (i) a simple PTM-classification method we developed based on semantic similarity calculations over the CellType ontology, and (ii) the results of the application of this method on an illustrative sample of 5 PTMs as a pre-requisite to highlighting the advantages and shortcomings of this method in the Discussion section.

**Figure 2:**
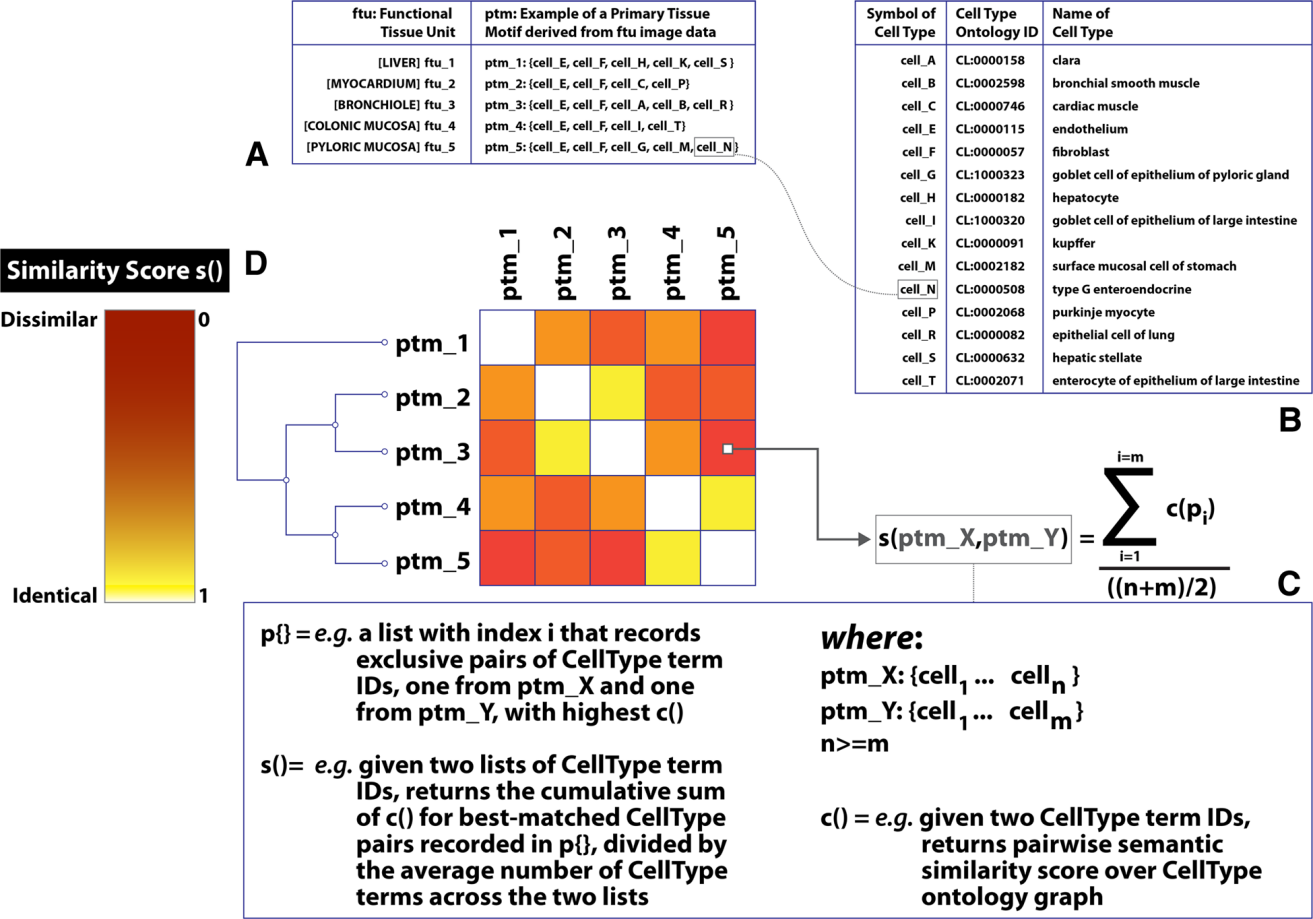
**Step-by-step example illustrating the automation of primary tissue motif comparison. [A]** FTU knowledge about 5 distinct tissues (in this particular example, derived from histology textbooks [16,17]) generated lists of distinct constituent cell types for each of the corresponding derivative primary tissue motifs. **[B]** Each distinct cell type in **[A]** was mapped to the equivalent term from the CellType ontology and assigned its unique term ID. **[C]** An all-*vs*-all pairwise comparison between the primary tissue motifs (*ptm*) was carried out as follows: (i) for every unique combination of *ptm* pairs (such that a pair consists of *ptm_X* and *ptm_Y*), an all-*vs*-all semantic similarity score **c()** for each unique combination of CellType term pairs is calculated (such that one CellType term is drawn from *ptm_X* and another from *ptm_Y*); (ii) the set **p{}** of highest scoring exclusive pairs of CellType terms is identified – exclusivity in a pair ensures that, once a CellType term from one *ptm* is selected to match another CellType term from another *ptm*, neither of these two CellType terms are included in any other pair in **p{}**; (iii) the sum of **c()** scores in **p{}** are divided by the average number of cell types across *ptm_X* and *ptm_Y* to generate **s(***ptm_X,ptm_Y***)**. **[D]** The set of *ptm* elements is clustered over the pairwise score **s()**. See also Table 1 for concrete values of **c()**, **p{}** and **s()** involving the 5 distinct tissues referred to in **[A]**.

Our method derives a pairwise similarity score **s()** for the comparison of cellular constituents in a primary tissue motif. The results of function **s()** provide the means to classify FTUs by clustering over this score. The key steps carried out in this method are exemplified below, by way of demonstration, and correspondingly illustrated in Figure 2:

1) Primary motif knowledge for five hypothetical FTUs, representing hepatic, cardiac, pulmonary, colonic and gastric tissue, was curated from histology accounts found in standard textbooks [16,17] (Figure 2A). The connection between motifs and well-defined anatomical location remains implicit here but, formally, the link can be readily registered using the anatomicalLocation relation introduced in the section above. The FTUs considered in the present case have distinct, but overlapping, primary motifs (in the sense of ptm-overlap, also discussed above);

2) The cellular constituents of the primary motifs were mapped to equivalent terms in the CellType ontology (Figure 2B) – this particular step involved 15 distinct CellType terms;

3) An all-*vs*-all semantic similarity calculation for the above 15 terms was carried out over the CellType ontology graph through the application of the approach described by Gentleman [18] (*the results of these calculations were courtesy of F. Couto, University of Lisbon*) to create a CellType similarity matrix c() (Figure 2C);

4) A PTM pairwise alignment algorithm was designed and implemented to compare the cellular constituents of two motifs. This step generates a list p{} of exclusive pairs of CellType terms, such that (i) a pair consists of a unique combination of two CellType terms (one from each respective motif) that have the highest possible score over c(), and (ii) a CellType term from a primary motif is involved in only one best-matched pair in p{}. The motif alignments, and the individual c() scores for each best-matched pair, for every possible pairwise comparison of motifs described in step 1 are listed in the last column of Table 1

5) An index of similarity s() between two motifs is then calculated by dividing (i) the cumulative summation of the c() scores for best-matched CellType pairs recorded in p{} by (ii) the average number of CellType terms across the two motifs. These scores are also listed in Table 1;

6) The set of 5 primary tissue motifs are subsequently clustered on the basis of their s() score to produce the topology of a binary tree illustrated in Figure 2D.

**Table 1:** **Results from pairwise primary tissue motif calculations in Figure**2

**No.**	**ptm**	**ptm**	**Score s()**	**p{} listing closest-matching cell pairs with corresponding c() scores**
**1.**	ptm_1	ptm_1	1.0	CL_0000182-CL_0000182:1.0, CL_0000632-CL_0000632:1.0, CL_0000057-CL_0000057:1.0, CL_0000091-CL_0000091:1.0, CL_0000115-CL_0000115:1.0
**2.**	ptm_1	ptm_2	0.64	CL_0000057-CL_0000057:1.0, CL_0000115-CL_0000115:1.0, CL_0000182-CL_0000746:0.29, CL_0000632-CL_0002068:0.25
**3.**	ptm_1	ptm_3	0.6	CL_0000057-CL_0000057:1.0, CL_0000115-CL_0000115:1.0, CL_0000182-CL_0000082:0.46, CL_0000632-CL_0000158:0.3, CL_0000091-CL_0002598:0.23
**4.**	ptm_1	ptm_4	0.64	CL_0000057-CL_0000057:1.0, CL_0000115-CL_0000115:1.0, CL_0000182-CL_0002071:0.3, CL_0000632-CL_1000320:0.24
**5.**	ptm_1	ptm_5	0.55	CL_0000057-CL_0000057:1.0, CL_0000115-CL_0000115:1.0, CL_0000182-CL_0000508:0.32, CL_0000632-CL_0002182:0.29, CL_0000091-CL_1000323:0.17
**6.**	ptm_2	ptm_2	1.0	CL_0002068-CL_0002068:1.0, CL_0000057-CL_0000057:1.0, CL_0000746-CL_0000746:1.0, CL_0000115-CL_0000115:1.0
**7.**	ptm_2	ptm_3	0.72	CL_0000057-CL_0000057:1.0, CL_0000115-CL_0000115:1.0, **CL_0002598-CL_0000746:0.64**, CL_0000082-CL_0002068:0.25
**8.**	ptm_2	ptm_4	0.6	CL_0000057-CL_0000057:1.0, CL_0000115-CL_0000115:1.0, CL_0000746-CL_0002071:0.22, CL_0002068-CL_1000320:0.17
**9.**	ptm_2	ptm_5	0.6	CL_0000057-CL_0000057:1.0, CL_0000115-CL_0000115:1.0, CL_0000508-CL_0000746:0.23, CL_0002182-CL_0002068:0.2
**10.**	ptm_3	ptm_3	1.0	CL_0000158-CL_0000158:1.0, CL_0000082-CL_0000082:1.0, CL_0000057-CL_0000057:1.0, CL_0002598-CL_0002598:1.0, CL_0000115-CL_0000115:1.0
**11.**	ptm_3	ptm_4	0.64	CL_0000057-CL_0000057:1.0, CL_0000115-CL_0000115:1.0, CL_0000158-CL_1000320:0.3, CL_0000082-CL_0002071:0.29
**12.**	ptm_3	ptm_5	0.57	CL_0000057-CL_0000057:1.0, CL_0000115-CL_0000115:1.0, CL_0000158-CL_0002182:0.35, CL_0000082-CL_0000508:0.31, CL_0002598-CL_1000323:0.21
**13.**	ptm_4	ptm_4	1.0	CL_1000320-CL_1000320:1.0, CL_0002071-CL_0002071:1.0, CL_0000057-CL_0000057:1.0, CL_0000115-CL_0000115:1.0
**14.**	ptm_4	ptm_5	0.71	CL_0000057-CL_0000057:1.0, CL_0000115-CL_0000115:1.0, **CL_1000323-CL_1000320:0.52**, CL_0002182-CL_0002071:0.33
**15.**	ptm_5	ptm_5	1.0	CL_0002182-CL_0002182:1.0, CL_0000508-CL_0000508:1.0, CL_0000057-CL_0000057:1.0, CL_1000323-CL_1000323:1.0, CL_0000115-CL_0000115:1.0

The unique combinations of primary tissue motif (**ptm**) pairs, discussed in Figure 2, are listed in the second and third columns. The last column shows the comma-delimited list of exclusive best-matched pairs in the following format: <CellType term ID from one ptm> - <CellType term ID from the other ptm> : <c()>.

The 5 PTMs were clustered into two tissue pairs and a hepatic out-group. The basis for the clustering of the two gastrointestinal PTMs can be found in the relatively high c() score of 0.52 between pyloric (CL_1000323) and colonic (CL_1000320) goblet cells, together with a more modest contribution of 0.33 from the pairing of gastric (CL_0002182) and colonic (CL_0002071) epithelial cells. The second cluster paired cardiac and bronchial PTMs on the strength of the shared presence of musculature which lead to a **c()** score contribution of 0.64 through the matching of smooth (CL_0002598) and cardiac (CL_0000746) muscle cells.

## Discussion

PTMs provide the list of constituent cell types in an FTU, as well as the anatomical location of these cells. The biophysical constraints satisfied by an FTU ensure that data about molecular secretions and surface receptors associated with the PTM’s list of cell types may be legitimately included in the realistic construction of the FTU’s molecular interaction network. In addition, the same lists of constituent cells provide a surrogate means by which FTU structures may be quantifiably compared, in a manner analogous to the application of primary amino acid sequence comparison in the comparison of complex 3D protein entities.

Below we discuss further work to be done in this area of tissue knowledge representation to address potential (a) limitations of the underlying theory of FTUs, (b) shortcomings with the availability of associated empirical data, as well as (c) oversimplifications in our classification approach.

### Theory

For a theory of transport physiology to be generally applicable, the concept of an FTU must factor in the presence of more than one advective channel in the organizational unit. In that sense, a theory of FTUs must be extended to represent different advective systems to be located within diffusion distance within the same FTU (*e.g.* as in epithelial cases such airway, renal, biliary and intestinal conduits). The vascular component of the FTU theory must also encompass the convective circulation of all types of extracellular fluid to include, for instance, plasma, lymph, tissue fluid, cerebrospinal fluid, synovial fluid, ocular humours and peritoneal fluid. Furthermore, FTUs must also account for two key properties of capillary networks, namely that:

1) any particular cell may be within diffusive range of more than one capillary (and therefore may be part of more than one FTU), and

2) given that, on average, a capillary is about 500 μm in length, compared to an approximately 60-μm stretch in an FTU, it is possible that the constitution of the FTU (and, therefore, that of its derivative primary motif) may alter along the course of the capillary from the arteriolar to the venular end.

### Data

The large scale cataloguing of FTU-related data is a key challenge in providing quantitative measures about the relative proportion and spatial distribution of cells along the FTU, as a first step to (a) predicting surface adhesion interactions that require cells to be in contact with one another, and (b) developing realistic mathematical simulations of tissue-level processes over a faithful geometric representation of the organization of cells in space.

Apart from the generation of significant quantities of volumetric FTU images, two key data shortcomings that stand in the way of realistic calculations about multi-organ molecular networks are:

(i) terminological data: given the complexity of cellular subtyping, current standard reference ontologies for cell types may not be sufficiently granular to meet tissue annotational requirements across the board (*e.g.* the January 2013 version of CellType ontology [19,20] (available via http://bioportal.bioontology.org) does not yet support the functionally critical distinction [21] between dermal fibroblasts of papillary and reticular origin);

(ii) biochemical data: crucially, not all substances suspended in tissue fluid or plasma are able to cross all types of endothelial lining and underlying basement membrane (*e.g.* the blood–brain barrier being one extreme case in point). The cataloguing of tissue-specific biochemical indices [22] for relevant extracellular substances (*e.g.* permeability, reflection co-efficient) would ensure a reliable calculation of sustainable molecular networks that cross the semi-permable capillary membrane.

It is anticipated that addressing the formidable obstacles outlined above will require community-level co-ordinated contribution to generate the requisite data and knowledge.

### Classification

While the overall classification method, applied to the 5 PTMs depicted in Figure 2, verifiably clusters tissues according to well-known structural and functional criteria (*e.g.* the shared presence of musculature in heart and bronchiole, and the closely-related epithelial features of the two gut mucosae) the overall approach is subject to limitations in terms of the precise biological meaning of PTMs and their automated comparison.

For instance, the curation of the 5 PTMs in our example may misleadingly suggest that all these PTMs have exactly the same type of endothelial and fibroblast cell within their corresponding FTUs (and subsequently classified on this basis). While this is unlikely to be the case in practice (*e.g.* it is not likely that the subtype of endothelial cell in the fenestrated liver circulation will be the same as that in the heart [23]), the lack of appropriate terms representing endothelial subtypes in the CellType ontology highlights the limitation of terminological granularity discussed above. In future, the application of semantic similarity calculations over gross anatomy terms linked to the PTM’s location knowledge may allay this representational shortcoming.

## Conclusion

**Figure 3:**
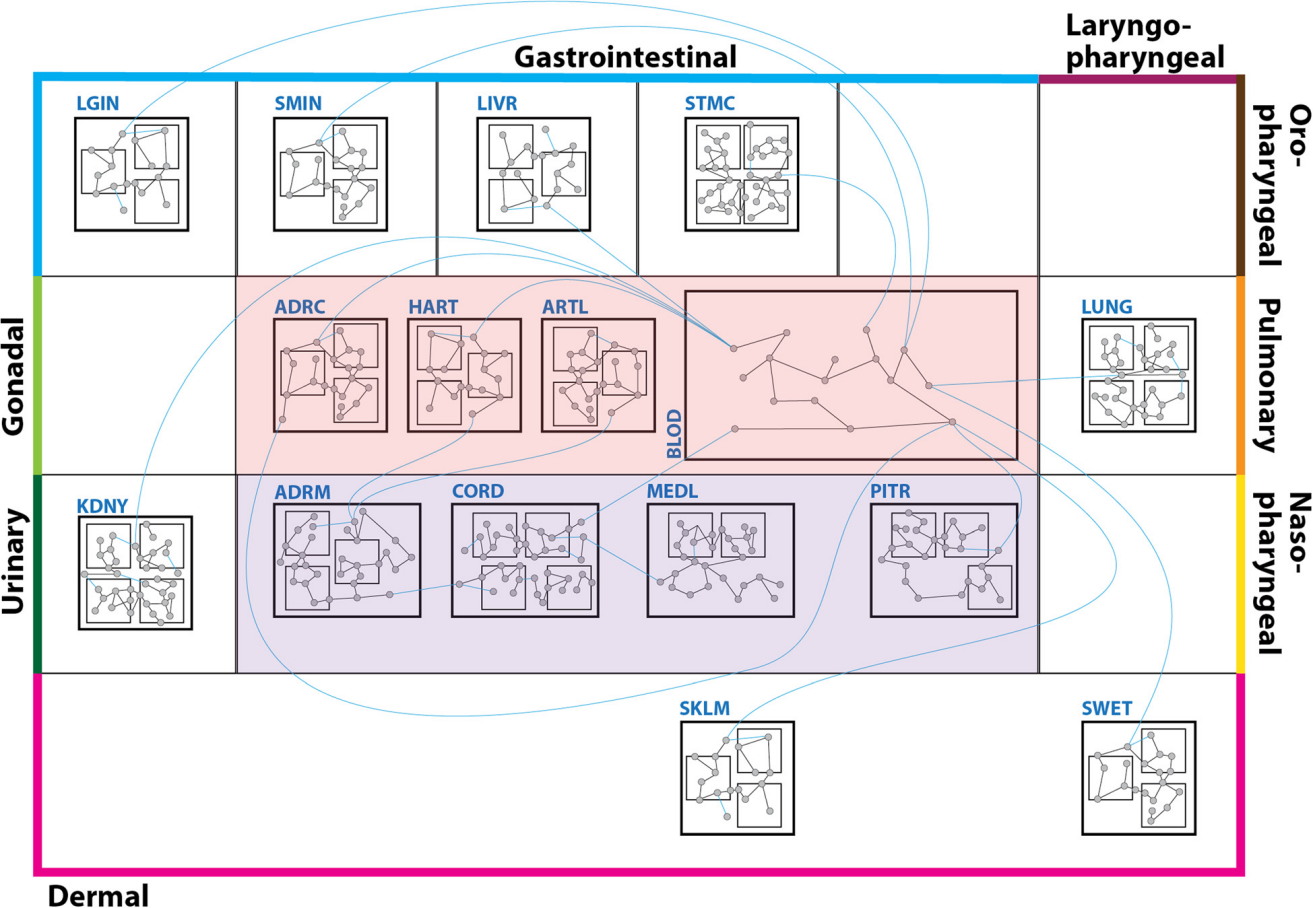
**Mock up of whole-body treemap schematic depicting a multi-organ endocrine pathway consiting of interlinked FTU-level molecular networks.** Figure 1D is extended to depict a number of primary tissue motifs representing FTUs involved in the endocrine regulation of electrolyte and blood pressure levels during exercise. Every individual motif is labeled (in blue – see below for key to label abbreviations). In this mockup of an ApiNATOMY [9] schematic, nesting of one box within another represents the *part_of* relation such that: (i) tiles representing motifs are nested within tiles representing the anatomical region of origin for the tissue material from which the FTU was acquired, and (ii) tiles representing the constitutent cells of the motif are nested within the corresponding motif tile. The position of nodes in the treegraph overlaid onto the treemap depicts the location of substances (*i.e.* molecules or charged atoms), with respect to the motif constituents, as follows: a node within the boundary of a cell tile represents an intracellular substance; on the boundary of a cell tile represent a molecule tethered to the plasma membrane of that cell; outside all cell boundaries represents a substance located in the extracellular tissue fluid of the corresponding FTU. TISSUE MOTIF LABELS: [LGIN: Large Intestine; SMIN: Small Intestine; LIVR: Liver; STMC: Stomach; ADRC: Adrenal Cortices; HART: Heart; ARTL: Arterioles; BLOD: Blood; LUNG: Lungs; KDNY: Kidneys; ADRM: Adrenal Medullae; CORD: Spinal Cord; MEDL: Medulla Oblongata; PITR: Pituitary; SKLM: Skeletal Muscles; SWET: Sweat Glands]. EDGE COLOUR: [Black: Molecular Binding; Blue: Intercompartmental Translocation].

An FTU is a unit of tissue organization where diffusive and advective flow transport modalities interlink, thus connecting molecular processes involving tissues or organs that are far apart (Figure 3). In our work, we outline the precise biophysical rationale for a rigorous definition of the FTU. In so doing, we acknowledge (i) the fundamental role of capillaries in directing and radically informing the formation of tissue architecture (*e.g.* as discussed in ref [24]), as well as (ii) the importance of taking into full account the critical influence of neighbouring cellular environments when studying complex developmental and pathological phenomena (*e.g.* the effect of stromal cells on cancer progression [25]).

By analogy to protein domain structure, which exhibits a backbone of linked alpha carbons tethering amino acid residues that interact to form a functional peptide unit, a capillary serves as an organizational backbone to an interacting cluster of cells that constitutes an FTU. The primary motif of an FTU coherently maps (i) terms representing these interacting cells to (ii) terms representing anatomical location. This mapping approach squarely addresses a major use case requirement in mammalian systems biology to computationally integrate data and model resources linked to biological structure across the divide of tissue scale. Given the increasing availability of resource metadata that are semantically annotated with terms from standard reference ontologies (*e.g.* as discussed in [26]), the potential generation of multi-tissue FTU catalogues opens an avenue for the functional bridging of cellular-level high throughput molecular resources with organ-level clinical resources.

The realistic description of molecular processes is at the core of the notion of FTUs and their derivative motifs. In future, we anticipate the development of a hierarchy of tissue motifs (a hierarchy analogous to that of primary, secondary, tertiary and quaternary protein structure descriptions) with which to organize the depiction of molecular processes. In this hierarchy, each motif level increase cumulatively provides more detail about the molecular interaction network held in the FTU. In this paper, for instance, we described the types of cell in a primary tissue motif that contribute secreted molecules and cell-surface receptors to this interaction network. It is envisaged that a secondary tissue motif will further enrich this network by taking into account those cells that are in direct plasma membrane contact with one another, enabling surface-to-surface molecular interaction (*e.g.* cell adhesion). Additional interactions brought into an FTU’s molecular network by advective means (*e.g.* through the blood supply) would then characterize the contribution of a tertiary tissue motif.

## Methods

### Part 1: Representing knowledge about primary tissue motifs

Maintaining a registry, classifying and recording characteristic knowledge about PTMs motivates the specification of a knowledge representation. We briefly overview the approach taken and elicit a number of requirements for such knowledge representation.

We adopt a formal ontological framework, the Basic Formal Ontology [10], to provide a general formal theory underlying the ontological analysis and formal treatment of FTUs and their derivative PTMs. BFO has been chosen for its simplicity and clear-cut, general distinctions such as, for example, between objects and the processes in which they participate. In practice, such provision allows for making domain specific ontologies more relevant to their intended domain (by off-loading generalities) and more structured (by adopting constraints from the adopted generic framework). For example, BFO makes provision for property-like entities that depend upon their bearers - this provision proves useful in conceptualising motifs, as we will see shortly. Formally, the distinction between functional tissue units and tissue motifs is enforced via the BFO distinction between higher-level categories of object-like entities and property-like entities. In the present work, the recourse to BFO thus allows us to secure, albeit here implicitly, the formal treatment of the general aspects of FTUs, TMs and their relations (categorical distinction, mereotopological features of FTUs, existential dependence of PTMs upon FTUs and so on). Furthermore, as the BFO framework has already been applied in related areas of the biomedical domain (in particular, in the context of the Open Biological and Biomedical Ontologies (OBO) Foundry [27]), this choice facilitates some degree of integration of the present treatment with related, existing or forthcoming, formalizations *ab initio*.

In BFO parlance, the world is made of two main kinds of things: objects, such as material objects and processes that involve these objects. We find this high-level dichotomy adequate for dealing with FTUs, their derivative motifs, and their role in physiology processes. According to this view, tissue motifs are on the side of objects insofar as these motifs are patterns of structural organization of possibly complex objects (*i.e.* tissues). But tissue motifs are of course not these objects: they are not tissues; they are repeating patterns of tissue structure. In BFO, entities such as patterns fall under a category of so-called *Generically Dependent Continuant* (which means that tissue motifs need some other entity in order to exist). Thus, we separate (i) motifs as entities in their own right from (ii) the entities (*i.e.* FTUs) in which they recur as patterns.

While these considerations solve the question of the ontological status of tissue motifs, they do little to provide the formal means for describing and registering the characteristics of tissue motifs in general and, in particular, for registering the differential characteristics between distinct motifs. Certainly, as generically dependent entities, tissue motifs can be characterized as the motifs of some tissue. This however does little more than secure a form of bookkeeping and, while it is fundamental for some purpose to identify and collect the association between tissues and their motifs, more detail is needed. One reason why such associations are important is that tissue motifs give a key to the classification of tissues. Furthermore, once the description of tissue motifs includes enough of the physical characteristics from which to derive characterizations of the physiological processes, they allow richer characterizations of tissues to be achieved, including characterizations of physiological processes now occurring at the tissue level of granularity.

The elements of the required tissue framework can separated into three main kinds according to the representation they support:

1) the characterization of tissue motifs through (i) the type of relationships in which they enter with other entities such as anatomical location, tissues and material parts of these tissues (*e.g.* cells and fluid compartments), as well as (ii) the way they are configured in virtue of presenting a given motif;

2) the elicitation of selected aspects of tissue motifs allowing for deriving the characteristics of the physiological processes they enable (spatial relationships and distances, in particular), as well as the various types of processes in question (*e.g.* processes of flow, stress transfer, electrical transmission, *etc.*);

3) the description of emerging biological properties and functions that tissues have in virtue of presenting given motifs or their combinations.

An interesting, and also challenging, aspect of such knowledge representation is that it brings together, through the central treatment of tissue motifs, treatments that are traditionally circumscribed to areas of the biomedical domain but that lack the required articulation to support a multi-scale ontology of transport physiology (*e.g.* as discussed in [28]). Given tissue motifs and their formal account, it is possible to articulate the description of transport phenomena from scales that range from the molecular to the organ level. Tissue motifs, therefore, provide a key bridge for the representation of transport physiology, which can now be traversed as a network of connected and interdependent knowledge representations.

The presentation of a full-fledged formalisation of the knowledge representation answering the above requirements is beyond the scope of this paper. Furthermore, our methodology is to adopt a dual bottom-up and top-down approach grounded in the experimentation with image annotation and their knowledge management. Thus a number of particular questions remain open at this stage and the formalisation outlined here is largely an item of future work. A preliminary specification of the core concepts is given in the Results section of this paper.

### Part 2: Acquiring and annotating FTU data

**Figure 4:**
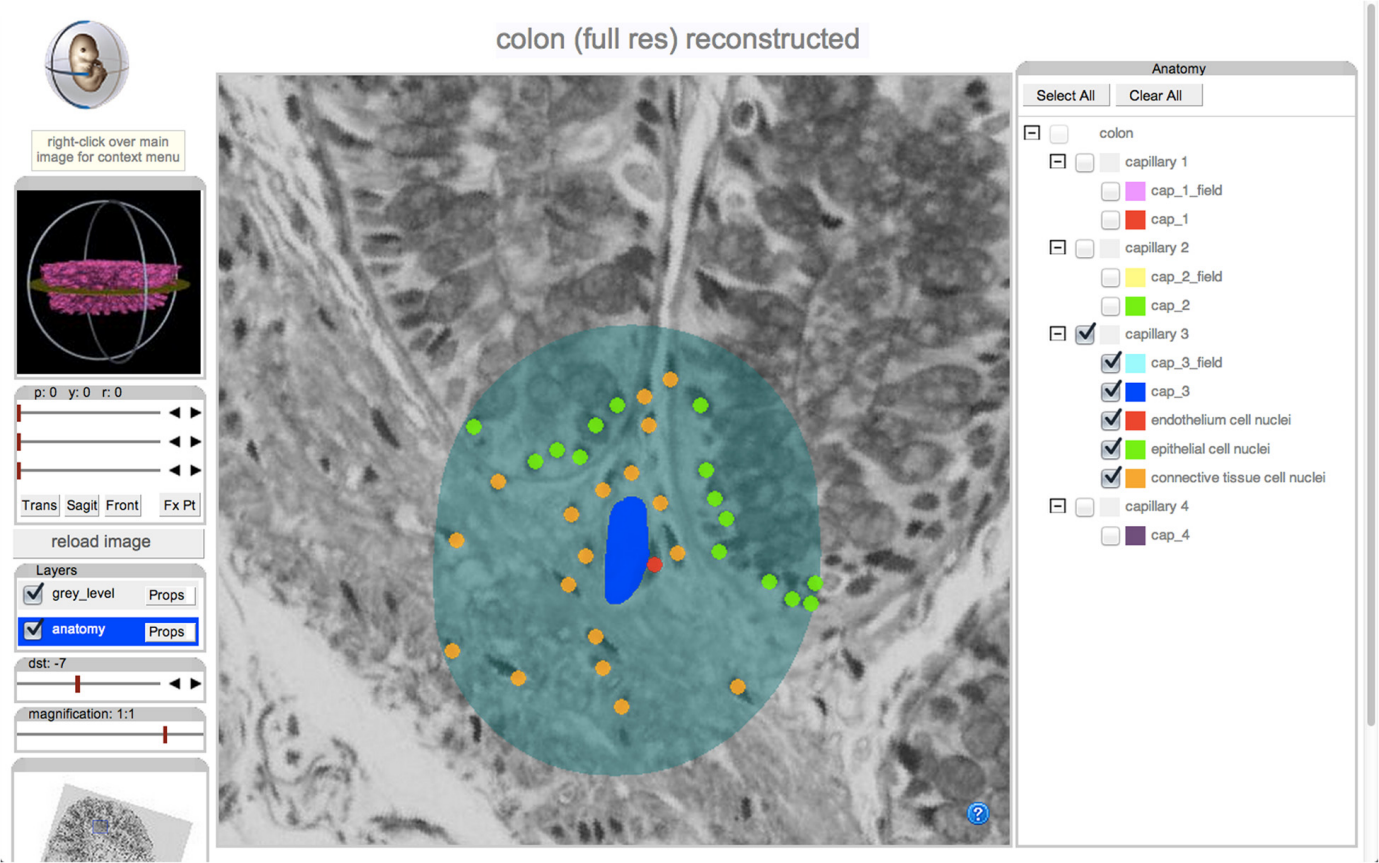
**Annotation of the location of cell nuclei within a vascular diffusive field.** Cellular nuclei within 40 μm of the centre of the vascular lumen (light blue shading) were identified and annotated onto three broad cellular categories, namely: 'endothelial’ (red dot), 'epithelial’ (green dot) and 'connective tissue’ (orange dot). The interface shown in this screenshot can be found at: [ http://aberlour.hgu.mrc.ac.uk/eAtlasViewer_demo/application/TPRDemo/wlz/colonRecon.php].

The prototypical histology image acquisition and annotation workflow required for FTU data production is exemplified in the process we specifically carried out to generate and annotate three-dimensional (3D) image data from human colon tissue (*tissue material provided by David Harrison, University of St. Andrews*). Following visual inspection of the reconstructed 3D image, by way of demonstration, the following sample annotations were generated for:

1) the location and course of small-calibre blood vessels (in view of the distortion caused by the plastic embedding procedure in this particular experiment, the positive identification of capillaries could not be conclusively ascertained). A movie that cycles through a set of contiguous slides illustrating the course of three colonic vessels, coloured red, green and blue, is available through the Additional file 1: Movie S1;

2) the approximate diffusive field surrounding a blood vessel was automatically calculated by drawing a 40-μm radius centred on the vascular lumen, in the plane of the original histological sections, using the image processing operation of dilation (as in this particular example the long axis of the vessel is not absolutely orthogonal to the plane of these sections, further refinement of our approach to diffusive field calculations is necessary). Additional file 2: Movie S2 shows the traversal - twice, in opposite directions - of an approximately 60-μm stretch along a vessel. This movie consists of two identical sets of 30 histology images - the slice thickness between on image and another being 2 μm. The first 15 secs of the movie shows the location of the vessel (in dark blue) over the 30 contiguous slides. The second 15-sec half of the movie traverses the same 30 slides in the reverse order showing both the central lumen and a 40-μm dilation that surrounds it;

3) the location of cellular nuclei within the approximate diffusive field. This step annotated cellular nuclei onto three broad cellular categories, namely: 'endothelial’ (Figure 4, red dot), 'epithelial’ (green dot) and 'connective tissue’ (orange dot). The full set of annotations over the 30 slides discussed above is presented in Additional file 3: Movie S3, and the result of their reconstruction is animated in 3D in Additional file 4: Movie S4.

The full dataset from this work (generated by RB and BdB) can be navigated at the following URL: [ http://aberlour.hgu.mrc.ac.uk/eAtlasViewer_demo/application/TPRDemo/wlz/colonRecon.php].

### Part 3: Ethical approval for experimental work on human tissue

Tissue collection took place in Edinburgh through the Pathology Department in Lothian University Hospitals. The laboratory is clinically accredited (under UK Clinical Pathology Accreditation programme).

The tissue collection was conducted under GLP standards (UK MHRA inspected) and subject to regulation and inspection by Scottish Government: Health Improvement Scotland (HIS) Team. A range of ethical permissions is in place (07/S1102/33: 08/S1103/10: 08/S1101/41: 10/S1402/33) granted by South East Scotland Ethics Committee. These permissions allow tissue and clinical data to be collected with full consent, but also permit use of unconsented tissue surplus to diagnostic requirement, providing that the tissue would have been removed anyway and the sample is completely anonymised. Permission includes use for academic research, teaching and training, provision to commercial companies and export overseas, including all microscopy techniques as well as molecular biology and proteomic uses, on completion of a simple Material Transfer Agreement. In this context, the work described in this paper adheres to operational principles that are consistent with the Oviedo convention and the Helsinki declaration in its last 2002 amendment. This ensures that appropriate consent has been obtained for the envisaged use of data, prior to anonymisation.

## Competing interests

The authors declare that they have no competing interests.

## Authors’ contributions

The work and the redaction of the paper is the result of the authors’ collaboration. BdB developed and curated the knowledge of functional tissue units and associated primary motifs, and carried out classification of the latter set. PG formulated the required characteristics of the knowledge representation presented in the Methods section. RB provided colon tissue image data and associated navigational tools. PH provided expertise in biophysics and in the quantification of FTU dimensions. BdB produced a first draft of the paper, which was subsequently collaboratively optimized by the authors. All authors read and approved the final manuscript.

## Supplementary Material

Additional file 1: Movie S1. Movie that cycles through a set of contiguous slides (2 microns apart) taken from human colon illustrating the course of three capillary-like vessels coloured red, green and blue. Click here for file

Additional file 2: Movie S2. Movie that follows in close-up the traversal - twice, in opposite directions - of an approximately 60-μm stretch along the blue colonic vessel shown in file 1. Click here for file

Additional file 3: Movie S3. Movie showing the location of cellular nuclei within the approximate diffusive field of a colonic blood vessel. The nuclei are annotated onto three broad cellular categories, namely: 'endothelial’ (red dot), 'epithelial’ (green dot) and 'connective tissue’ (orange dot). Click here for file

Additional file 4: Movie S4. 3D animation of the annotation reconstruction for a colonic FTU. Click here for file
